# Dysbiosis of vaginal and cervical microbiome is associated with uterine fibroids

**DOI:** 10.3389/fcimb.2023.1196823

**Published:** 2023-09-06

**Authors:** Xuetao Mao, Hao Chen, Xuan Peng, Xingping Zhao, Zheng Yu, Dabao Xu

**Affiliations:** ^1^ Department of Gynecology, The Third Xiangya Hospital, Central South University, Changsha, China; ^2^ Department of Parasitology, School of Basic Medical Science, Central South University, Changsha, China; ^3^ Department of Microbiology, School of Basic Medical Science, Central South University, Changsha, China

**Keywords:** uterine fibroids, vaginal microbiome, cervical microbiome, dysbiosis, microbial interaction

## Abstract

Dysbiosis of the female reproductive tract is closely associated with gynecologic diseases. Here, we aim to explore the association between dysbiosis in the genital tract and uterine fibroids (UFs) to further provide new insights into UF etiology. We present an observational study to profile vaginal and cervical microbiome from 29 women with UFs and 38 healthy women, and 125 samples were obtained and sequenced. By comparing the microbial profiles between different parts of the reproductive tract, there is no significant difference in microbial diversity between healthy subjects and UF patients. However, alpha diversity of UF patients was negatively correlated with the number of fibroids. Increased Firmicutes were observed in both the cervical and vaginal microbiome of UF patients at the phylum level. In differential analysis of relative abundance, some genera were shown to be significantly enriched (e.g., *Erysipelatoclostridium*, *Mucispirillum*, and *Finegoldia*) and depleted (e.g., *Erysipelotrichaceae* UCG-003 and *Sporolactobacillus*) in UF patients. Furthermore, the microbial co-occurrence networks of UF patients showed lower connectivity and complexity, suggesting reduced interactions and stability of the cervical and vaginal microbiota in UF patients. In summary, our findings revealed the perturbation of microbiome in the presence of UFs and a distinct pattern of characteristic vaginal and cervical microbiome involved in UFs, offering new options to further improve prevention and management strategies.

## 1 Introduction

Uterine fibroids (UFs), also known as uterine leiomyomas or myomas, are the most common nonmalignant neoplasms of the reproductive tract among reproductive-age women and are composed of smooth muscle cells and fibroblasts (Stewart, 2001; Stewart et al., 2016). It is estimated to affect more than 25% of women worldwide, particularly black women (Baird et al., 2003). Careful pathological examination of hysterectomy histopathologic specimens showed that the incidence of UFs was as high as 77% (Cramer and Patel, 1990; Harris et al., 2022). Although UFs are often asymptomatic, reasonable therapy is still needed for many women who have significant symptoms and consequences that include heavy or prolonged bleeding, anemia, pelvic pain, even infertility, and adverse pregnancy outcomes (Donnez and Dolmans, 2016; Zepiridis et al., 2016). In addition to the significant impacts on quality of life, UFs also incur heavy individual and societal costs, including surgery, hospital admissions, outpatient visits, psychological distress, and medications (Al-Hendy et al., 2017; Millien et al., 2021). The pathophysiology of UFs is still under investigation, including genetic susceptibility, sex steroid hormones, and abnormal stem cell transformation (Moravek et al., 2015; El Sabeh et al., 2021).

The human microbiome has co-evolved with the human as a unity called holobiont (Postler and Ghosh, 2017). The microbial communities play a fundamental role in human health and diseases, performing key functions in digestion, metabolism, mood and behavior, development and immunity, and a range of acute and chronic disorders (Ottman et al., 2012). Furthermore, the existence and invasion of microbiota inhabited along the female reproductive tract have long been known to impact female reproductive health and the onset of gynecological diseases (e.g., gynecological cancers, infertility, preterm birth, polycystic ovary syndrome, cervical intraepithelial lesions, and/or endometriosis) (Nieves-Ramirez et al., 2021; Salliss et al., 2021; Zhu et al., 2022). Generally, the vaginal microbiome in healthy women is dominated by *Lactobacillus*, but this ecosystem could be disrupted in the presence of some vaginal microorganisms, such as *Atopobium vaginae*, *Mycoplasma*, and *Prevotella bivia*, as well as a decrease in the proportion of *Lactobacillus* spp. Exogenous pathogen infections, such as parasites (*Trichomonas vaginalis* and *Schistosoma*) and viruses (HPV, HIV, and HSV), also alter the microbiota of the female reproductive tract by influencing the host’s immune response and metabolism (Martin et al., 2013; Lebeau et al., 2022; Bongiorni Galego and Tasca, 2023). Given that a vast community of indigenous microorganisms colonize the reproductive tract and interact with the host in a symbiotic relationship, the microbiome also can alter among populations depending on the host’s diet, ethnicity, geographical environment, health status, etc. (Gupta et al., 2017; Song et al., 2020).

Previous studies demonstrated that the reproductive tract microbiota was involved in reproductive tract diseases through multiple approaches, including local immune responses and metabolic regulation, and we hypothesized that the reproductive tract microbiota might play a role in UFs. Emerging evidence suggests the dysbiosis of the female genital tract (FGT) microbiome in patients with UFs, while these findings are not comprehensive and uniform among studied populations. Prior clinical studies that used self-reported questionnaire data showed an association between an increased risk of fibroids and bacterial vaginosis (BV). BV is characterized by vaginal microbial community alteration in which the microbiome normally dominated by *Lactobacillus* switches to anaerobes like *Gardnerella vaginalis* (Moore et al., 2015; Moore and Baird, 2017). However, qPCR analysis of BV-associated bacteria and *Lactobacillus* found no powerful evidence to support the hypothesis that BV could increase the risk of leiomyoma incidence or growth in subsequent prospective studies (Moore et al., 2021; Moore et al., 2022). Lately, a study employing high-throughput 16S rRNA taxonomic profiling found that *Lactobacillus* spp. were more abundant in the vaginal and cervical samples of individuals without UFs, while *Lactobacillus iners* was more abundant in the cervix of UF patients (Chen et al., 2017). Moreover, the abundance and diversity of vaginal bacterial taxa were significantly higher in recurrent vaginitis patients with underlying uterine diseases (UFs, adenomyosis, and endometrial polyps) than those without (Kim et al., 2022). In summary, there are still some limitations in these studies of the correlation between reproductive tract dysbiosis and UFs, including confounding factors, lack of suitable controls, and insufficient sample size, so there is no consistent conclusion on the relationship between microecological imbalance in the reproductive tract and UFs, and further studies are necessary.

In this work, we collected 125 specimens from 29 patients with UFs and 38 healthy subjects to analyze vaginal and cervical microbiome. By comparing the microbial profiles between UF patients and healthy individuals, we revealed the perturbation of the genital tract microbial community in the presence of UFs. Moreover, we highlighted the pivotal role of genital tract microbial interplay in patients with UFs and healthy women.

## 2 Materials and methods

### 2.1 Study design and sample collection

Samples from the vagina and cervix of patients with UFs and healthy people were taken for this experiment during the same period. Volunteers who were not in the pre- or post-menopausal period were recruited between December 2020 and May 2021 at the Third Xiangya Hospital of Central South University. Patients with UFs all met clinical diagnostic criteria and all subjects underwent ultrasound within 1 month to identify the presence/absence of UFs (Stewart, 2015). None of the participants received recent vaginal medication or cervical treatment or had performed douching within the 7 days before sample collection. Exclusion criteria include the following: administration of exogenous estrogens, progestins, and antibiotics within 3 months; history of endocrine or autoimmune disorders, gynecological cancer, and adenomyoma; pregnant and lactating women; episodes of vaginitis and pelvic inflammatory disease (PID); HPV infection of the genital tract; and placement of intrauterine device (IUD). The samples were collected by swabs, including leucorrhea (drawn from the mid-vagina), and cervical mucus (drawn from the cervical canal) in the follicular phase of the menstrual cycle. The collected fresh samples were immediately stored in sampling tubes with the preservative solution and stored at −80°C until further processing.

For experiments involving human swab samples whose donors were identifiable, written informed consent was obtained from each study participant, according to protocols approved by the Institutional Review Board (IRB) of Third Xiangya Hospital, Central South University (under permit number 22224).

### 2.2 DNA extraction

Total genomic DNA was extracted with the OMEGA Soil DNA Kit (M5636-02) (OMEGA Bio-Tek, Norcross, GA, USA) according to the following steps. Weigh 500 mg of glass beads and 0.25–0.5 g of sample in a 2-ml centrifuge tube, add 0.7 ml of Buffer SLX Mlus and 70 μl of Buffer DS, and mix by vortexing. Then, incubating at 70°C for 10 min and centrifuging at 13,000×*g* for 5 min were performed. After transferring 500 µl of the supernatant into new 2-ml tubes and adding 170 µl of Buffer SP2, add 170 µl of HTR Reagent to samples and mix thoroughly by vortexing for 10 s. After incubating on ice for 5 min and centrifuging at 13,000×*g* in a microcentrifuge for 5 min, we transferred 450 µl of the cleared supernatant to new 1.5-ml tubes, added 450 μl of Buffer XP5 and 40 μl of MagSi Particles to the samples, and shook 60 s to mix well. Then, incubate at room temperature for 2 min and place the tube or plate on a magnetic separation device suitable for 2-ml tubes to magnetize the MagSi particles. We added 500 µl of Buffer XP5 to the test tube, then placed the tube on a magnetic separation device and carefully removed and discarded the cleared supernatant. After adding 800 μl of Buffer PHB into the tube, place the tube onto the magnetic separation device and carefully remove and discard the cleared supernatant. We added 800 μl of SPM Wash Buffer diluted with ethanol into the tube, placed the tube onto the magnetic separation device to magnetize the MagSi particles, and carefully removed and discarded the cleared supernatant. Wash MagSi particles with SPM one more time. After removing the supernatant, air-dry the magnetic beads by inverting the tube on absorbent paper for 15 min. Remove any residue liquid from the tube with pipettor. After adding 50–100 μl of Elution Buffer or water to the tube, incubate the tube and resuspend MagSi particles by vortexing at 65°C for 10 min. We transferred the cleared supernatant containing purified DNA to new 1.5-ml tubes and stored it at −20°C before analysis. The quantity and quality of extracted DNA were measured respectively using a NanoDrop NC2000 spectrophotometer (Thermo Fisher Scientific, Waltham, MA, USA) and agarose gel electrophoresis.

### 2.3 16S rRNA gene amplicon sequencing

PCR amplification of the V3–V4 region of the bacterial 16S rRNA gene was performed using the forward primer 338F (5’-ACTCCTACGGGAGGCAGCA-3’) and the reverse primer 806R (5’-GGACTACHVGGGTWTCTAAT-3’). Sample-specific 7-bp barcodes were integrated into the primers for multiplex sequencing. The components of PCR contained 5 μl of buffer (5×), 0.25 μl of fast Pfu DNA polymerase (5 U/μl), 2 μl (2.5 mM) of dNTP, 1 μl (10 μM) of each forward and reverse primer, 1 μl of DNA template, and 14.75 μl of ddH_2_O. Thermal cycling consisted of initial denaturation at 98°C for 5 min, followed by 25 cycles including denaturation at 98°C for 30 s, annealing at 53°C for 30 s, extension at 72°C for 45 s, and final extension at 72°C for 5 min. PCR amplicons were purified with Vazyme VAHTSTM DNA Clean Beads (Vazyme, Nanjing, China) and quantified using the Quant-iT PicoGreen dsDNA Assay Kit (Invitrogen, Carlsbad, CA, USA). After the individual quantification step, amplicons were pooled in equal amounts, and pair-end 2×250 bp sequencing was performed using the Illumina NovaSeq platform with NovaSeq 6000 SP Reagent Kit (500 cycles) at Shanghai Personal Biotechnology Co., Ltd (Shanghai, China).

### 2.4 Sequence analysis

After sequencing, the reads were de-multiplexed into samples according to the barcodes and the sequence was imported to the QIIME2 (version 2022.2) (Bolyen et al., 2019). The raw data were filtered to eliminate the adapter pollution and low-quality reads to obtain clean reads. Sequences were clustered at the 97% similarity level using the Vsearch plugin (Rognes et al., 2016). Taxonomic classifiers use classify-consensus-blast of the plugin feature-classifier (Camacho et al., 2009), based on the Silva 138 reference sequence (MD5: a914837bc3f8964b156a9653e2420d22) and taxonomy files (MD5: e2c40ae4c60cbf75e24312bb24652f2c) (Holgersen et al., 2021). Non-bacterial sequences and mitochondrial chloroplast contamination were removed by the taxa plugin.

### 2.5 Statistical analysis

All statistical analyses were performed by using the R environment (V4.2.1) (R Core Team, 2022). The statistical results were visualized using the “ggplot2” package without special instructions (Wickham and Wickham, 2016). The package “vegan” was used to calculate alpha diversity based on flat taxonomy table and obtain the differential expression matrix and the *p*-value matrix of bacterial composition at the genus level (Oksanen et al., 2022). The Gini–Simpson diversity index was obtained by subtracting the value of the classical Simpson index from 1. Beta diversity was analyzed using constrained principal coordinate analysis (CPCoA) by the “amplicon” package (Zhang et al., 2019; Liu et al., 2021), and bacterial community composition across all samples was based on Bray–Curtis distances. Venn was performed by the “ggvenn” package (Yan, 2021). Differential analysis of the relationship between the number of fibroids and Gini–Simpson was performed by the function “summary” in the R environment. Phylum-level Manhattan plot was computed using the “edgeR” package based on taxonomic information, and the *p*-value was corrected by the Benjamin and Hochberg false discovery rate (FDR) (Robinson et al., 2010). The taxonomic composition of microbiota at the phylum and genus level was completed using the “ggplot2” package. The package “DESeq2” was used to analyze abundance difference and marker genus based on the rules that the significance level was adjusted *p*-value < 0.05 and absolute foldchange value was greater than 1 (Love et al., 2014). The co-occurrence networks of microbiome within four groups were performed using the relative abundance table of genus level, and it was established based on Spearman correlation matrix and corrected *p*-value matrix using the “igraph” package (Csardi and Nepusz, 2006). FDR was used to correct the *p*-value. Genera with relative abundance lower than 0.1% were filtered out before analysis, and the thresholds of Spearman correlation coefficient and corrected *p*-values were 0.8 and 0.05, respectively. Network topology properties and hub networks were calculated using Gephi software (Bastian et al., 2009). Properties of the co-occurrence networks of microbiome were compared by Mann–Whitney *U* test in R environment.

Clinical data analyses were performed using SPSS 26.0 software (IBM Corp, Armonk, NY, USA). Continuous variables were presented as median with range (minimum–maximum) or mean ± standard deviation (SD) and appropriately analyzed with the Wilcoxon or *t* test. Categorical variables were described as percentages and evaluated using the Chi-squared or Fisher exact test. In addition, the Mann–Whitney *U* test was used to compare the differences in nonparametric data between the groups. *p*-value less than 0.05 (2-sided) was considered statistically significant.

## 3 Results

### 3.1 Demographic characteristics of the study population

In total, 29 patients with UFs and 38 healthy subjects were enrolled in this study. All participants were fully informed about the study protocol. The clinical information of the participants, including age, body mass index (BMI), menstrual history, and reproductive history, is shown in **Table 1**. No statistical significance for these characteristics was found between UFs and the healthy group. In addition, the clinical characteristics of the UF patients are also shown in **Supplementary Table S1**.

**Table 1:** Characteristics of the study subjects.

Variables	Control group (*n* = 38)	Uterine fibroid group (*n* = 29)	*p*-value
**Age [years, median (range)]**	34 (23–54)	40 (24–49)	0.065
**BMI [kg/m^2^, (mean ± SD)]**	22.6 ± 3.60	22.7 ± 3.38	0.923
**Number of gravidities**	2 (0–8)	3 (0–7)	0.355
**Number of parities**	1 (0–3)	1 (0–4)	0.803
**Number of abortions**	1 (0–5)	1 (0–5)	0.389
**Caesarean section delivery, *n* (%)**	9 (23.7%)	6 (20.7%)	0.771
**Menstruation [day, median (range)]**			
Duration	7 (5–13)	7 (4–13)	0.072
Frequency	28 (22–40)	28 (25–45)	0.079
**Increased flow volume, *n* (%)**	19 (50.0%)	9 (31.0%)	0.119

BMI, body mass index; SD, standard deviation.

### 3.2 The microbial diversity of the female genital tract

To explore whether the microbiome in the genital tract was altered in patients with UFs, we collected samples from the vagina and cervix. We collected a total of 50 samples from patients with UFs and 75 samples from healthy people. We divided samples into four groups according to different sampling sites in the genital tract and whether they had UFs: healthy individuals’ vaginal microbiome (HV, *n* = 38), UF patients’ vaginal microbiome (FV, *n* = 29), healthy women’s cervical microbiome (HC, *n* = 37), and cervical microbiome of patients with UFs (FC, *n* = 21) (**Supplementary Figure S1**). Upon analysis, the rarefaction curves of all samples were parallel to the *X*-axis (**Supplementary Figure S2**), indicating that all samples were sequenced to sufficient depth for subsequent analysis.

**Figure 1:**
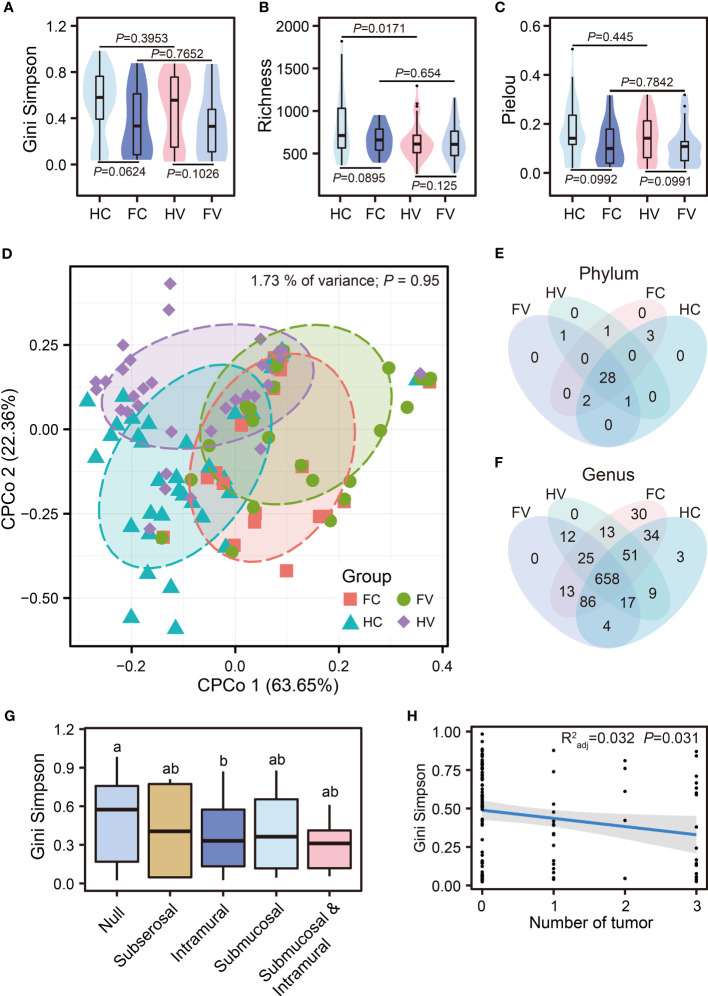
The diversity of microbial communities. Analysis of alpha diversity was presented by Gini–Simpson **(A)**, Richness **(B)**, and Pielou **(C)** among four groups. Differential analysis was performed by the Wilcoxon test. **(D)** Beta diversity was presented by CPCoA (Constrained Principal Coordinate Analysis) analysis based on Bray–Curtis distance. The difference of composition in phylum **(E)** and genus **(F)** levels between four groups. **(G)** Comparison of Gini–Simpson among different locations of tumor occurrence. The letters such as “a” and “b” indicate whether there is a significant difference between different groups. The same letters indicate no significant difference between different groups (Kruskal–Wallis test). **(H)** Regression analysis of tumor numbers and Gini–Simpson.

We performed analysis of alpha diversity in four groups to present the changes of numbers, abundance, and evenness of the microbiome in the genital tract. The results of the Gini–Simpson index showed that the alpha diversity of UF patients was lower than that of healthy people at different sampling points, but there was no significant difference **(** **Figure 1A** **)**. The Pielou index also showed no difference, suggesting no significant difference in the evenness of OTUs among the four groups. **(** **Figure 1C** **)**. Interestingly, we observed a significant difference in the Richness index between the groups of healthy people sampled from the vagina and cervix, respectively (*p* = 0.0171) (**Figure 1B**). We then compared beta diversity based on Bray–Curtis distances between the groups of patients and healthy people using constrained principal coordinate analysis (CPCoA) and found that there was no significant difference in Bray–Curtis distances among four groups, suggesting that the compositional differences of samples from four groups were relatively similar **(** **Figure 1D**). In addition, the relationship between alpha diversity and clinical variables (location and number of fibroids) was explored. No difference in the Gini–Simpson index was found in UF patients at different locations **(** **Figure 1G** **)**. The number of tumors showed a strong negative correlation with the Gini–Simpson index of vaginal and cervical microbiome in patients with UFs (*R*_adj_^2 = ^0.032, *p* = 0.031, **Figure 1H**). As the number of tumors increased, the alpha diversity decreased significantly.

### 3.3 The composition and biomarkers of the female reproductive tract

To explore alterations in the composition of bacterial communities located in different sites of the genital tract, we compared the differences in bacterial abundances among the four groups at the phylum and genus levels.

**Figure 2:**
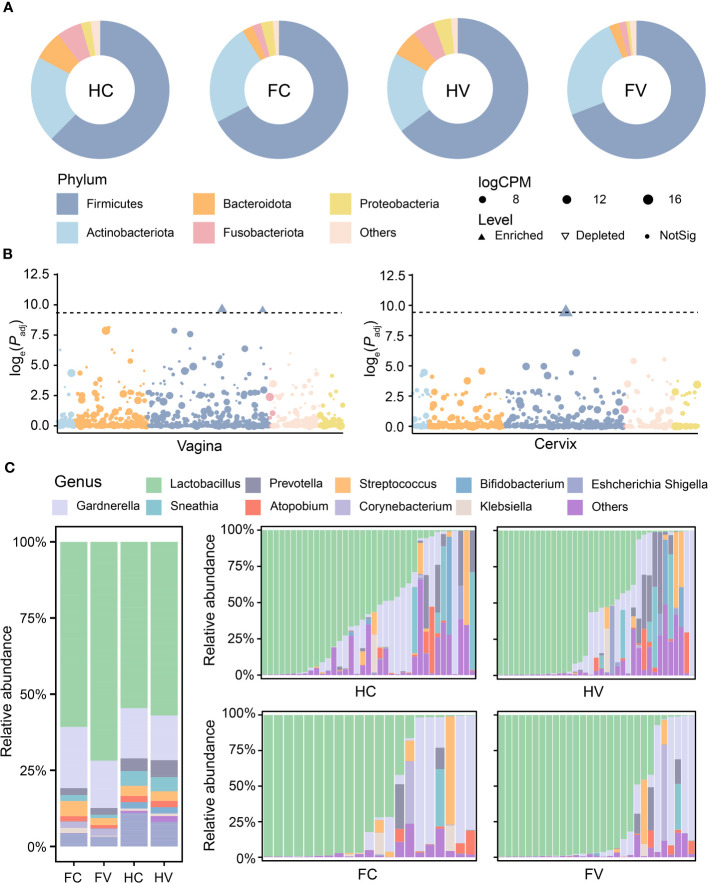
The composition of microbiome at the phylum and genus levels. **(A)** Microbial composition of four groups at the phylum level. **(B)** The difference analysis of microbiome in relative abundance at phylum levels between healthy and UF groups in the position of vagina and cervix. Dashed lines show that the threshold of *p*-value is 0.0001. Dots show that there is no significant difference in the relative abundance of phylum. Upwards filled triangles represented significant enrichment and downwards hollow triangles represented significant depletion in the relative abundance of phylum. **(C)** Microbial composition of four groups at the genus level.

The bacterial composition of the four groups had both commonalities and differences. At the phylum level, there were 31 phyla in the HV group, 32 phyla in the FV group, 33 phyla in the HC group, and 34 phyla in the FC group (**Figure 1E**). At the genus level, there were 785 genera in the HV group, 815 genera in the FV group, 862 genera in the HC group, and 910 genera in the FC group, of which 658 genera were common to all groups (**Figure 1F**). The differences in the bacterial genus level composition of the four groups were greater than at the phylum level. To further specify the bacterial differences, we then analyzed the composition of the flora at the phylum and genus levels. From the results of the phylum, the four groups were dominated by Firmicutes (**Figure 2A**). We analyzed bacterial alterations at the phylum level in UF patients and healthy people. Compared with the groups of healthy people, the abundance of Firmicutes showed a highly significant upward trend in the groups of UF patients (*p* < 0.0001) (**Figure 2B**). According to the compositional analysis at the genus level, *Lactobacillus* spp. was the dominant bacterium in the vagina and cervix (**Figure 2C**).

**Figure 3:**
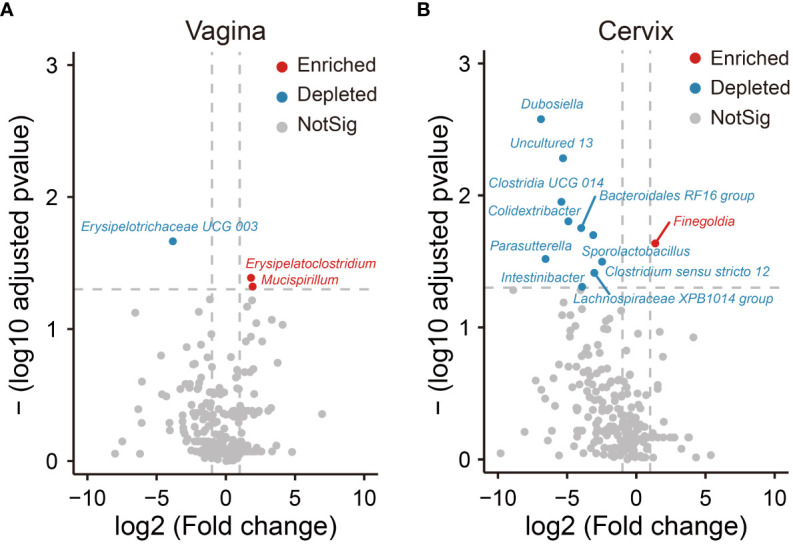
Differential analysis of relative abundance at the genus level. The analysis of volcano shows relative abundance of genera, which is significantly enriched and depleted in the UF groups compared to the healthy groups in the position of vagina **(A)** and cervix **(B)**. Dots in red show that the relative abundance of genera in UF groups enriched significantly compared to healthy groups. Dots in blue show that the relative abundance of genera in UF groups depleted significantly compared to healthy groups. Dots in gray show that there is no significant difference in relative abundance of genera between UFs and healthy groups.

In addition, we analyzed differentially abundant bacteria in the vagina and cervix based on diversity of biomarker signature differences. Compared to the group of healthy people, the volcano plot showed that one biomarker was found to be depleted (marked by blue) and two were enriched (marked by red) in patients with UFs in the vagina (**Figure 3A**). In the cervix, 11 distinct genera were found in the UF group relative to the healthy control, with 1 genus (highlighted in red) upregulated and 10 genera downregulated (highlighted in blue) (**Figure 3B**).

### 3.4 Microbial interactions and networks between microbiome in the female genital tract

**Figure 4:**
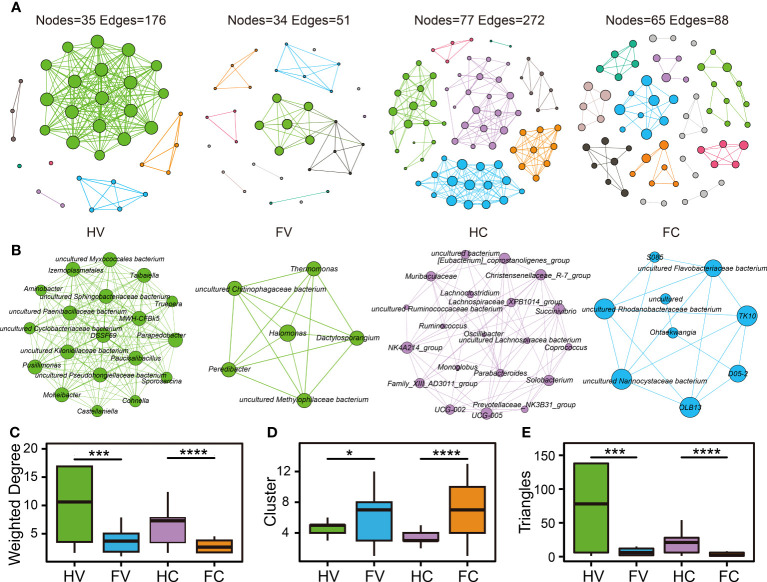
The co-occurrence network of microbiome in the four groups. The co-occurrence network **(A)** and hub network **(B)** of health and UF groups in the position of vagina and cervix. Comparison of network topology properties among four groups, containing weighted degree **(C)**, cluster **(D)**, and triangles **(E)**. Differential analysis was performed by Kruskal–Wallis test. *p < 0.05, ***p < 0.001, ****p < 0.0001.

We performed co-occurrence networks analysis to reveal the relationships among microorganisms. Based on the same network construction parameters, the network showed 35 nodes and 176 edges for the HV group, 34 nodes and 51 edges for the FV group, 77 nodes and 272 edges for the HC group, and 65 nodes and 88 edges for the FC group (**Figure 4A**). We analyzed the network properties for each group of networks. The microbial average weighted degrees in the UF patients and healthy people showed significant differences **(** **Figure 4C**). The number of cervical and vaginal triangles was also significantly lower in the UF groups (**Figure 4E**), suggesting that the connectivity and complexity of the genital tract microbiota were significantly lower in patients with UFs. In the analysis of clusters, clusters of vagina and cervix were significantly higher in the UF groups, which indicated that the average “clustering property” of the whole microbial network in both the vagina and cervix was higher in UF groups than in the healthy groups (**Figure 4D**). Meanwhile, we extracted and presented hub networks of four microbiomes with the highest degree of clustering by group (**Figure 4B**) to gain insight into each network in the UFs and healthy groups at different locations. Overall, the hub networks of cervical microbiome in healthy people had the highest number of nodes and aggregation.

## 4 Discussion

In this study, we conducted microbial profiling for cervical and vaginal microbiome associated with UFs, and further highlighted the interplay between microbial communities. By comparing the microbial profiles between different parts of the reproductive tract among groups, we revealed the perturbation of microbial community in the presence of UFs. It was found that microbiome of vagina and cervix in patients with UFs were altered in composition and ecological network compared with healthy women. The alpha and beta diversities showed no statistical significance between UF patients and the healthy women. However, a significant negative correlation was observed between Gini–Simpson and the number of fibroids. Moreover, higher microbial Richness index was found in the HC group than the HV group. Samples from patients with UFs exhibited significant alterations in Firmicutes at the phylum level. In differential abundance analysis, enriched *Erysipelatoclostridium*, *Mucispirillum*, and *Finegoldia* and depleted *Erysipelotrichaceae* UCG-003 and *Sporolactobacillus* were observed in UF groups. Furthermore, more dispersed and lower node degree distribution were presented in the networks of the UF groups (both in the vagina and cervix), suggesting lower connectivity and complexity than the networks of healthy groups.

In our study, the microbial alpha and beta diversities of vaginal and cervical microbiome between UF patients and healthy women were not significant (*p* > 0.05). These results were also in line with previous research on female reproductive disorders, which found that endometriosis, intrauterine adhesion, and cervical intraepithelial neoplasia showed no alteration compared with healthy people (Ata et al., 2019; Lin et al., 2022; Liu et al., 2022). Interestingly, the alpha diversity (Gini–Simpson index) of the microbiome was negatively correlated with the number of fibroids according to our results. The correlation between microbial imbalance and increase in the number of tumors indicated an important role of microbiota in the development of disease. For example, gut dysbiosis-stimulated cathepsin K secretion mediated TLR4-dependent M2 macrophage polarization and promoted tumor metastasis in colorectal cancer (Li et al., 2019). However, further experiments are needed to verify and explore possible mechanisms in benign UFs. Additionally, higher microbial Richness was observed in the healthy vagina than in the cervix, representing a unique ecological niche in cervical canal, as indicated by the heterogeneity of microbiota between this location and that of the vagina. On the other hand, highly cited research showed distinct biomass between vagina and cervix by qPCR test of *Lactobacillus* species, although no obvious difference was observed in microbial diversity (Chen et al., 2017).

Some gynecological diseases or organ malfunctions are marked by the presence of potential pathogenic microbes, whereas others are characterized by depletion of health-associated bacteria (Onderdonk et al., 2016; Brusselaers et al., 2019; Laniewski et al., 2020). In the vaginal microbiome, *Erysipelatoclostridium* and *Mucispirillum* were significantly enriched, while the *Erysipelotrichaceae* UCG-003 was depleted in UF patients. *Erysipelatoclostridium*, an opportunistic pathogen reported to be enriched in patients with allergic diseases and metabolic syndrome, modulates small intestinal glucose and lipid transport by altering intestinal barrier permeability (Woting et al., 2014; Shen et al., 2019; Labarta-Bajo et al., 2020; Li et al., 2022). *Mucispirillum*, observed more abundant in progression of hepatocellular carcinoma in mice driven by high cholesterol, may be reversed with atorvastatin treatment through bile acid biosynthesis pathway (Zhang et al., 2021). Moreover, *Erysipelotrichaceae* UCG-003 is one of the main bacteria that produce butyrate, which is considered to play an important role in maintaining the integrity of the colonic epithelium. The abundance of *Erysipelotrichaceae* UCG-003 was found to be decreased in patients with neurological disorders and lung cancer compared with healthy individuals (Singh et al., 2019; Zhao et al., 2021; Kaiyrlykyzy et al., 2022). Regarding the cervical microbiome, *Finegoldia* usually appears on the skin and mucous membranes and is associated with vaginosis, as well as infectious diseases and soft tissue abscesses. More importantly, *Finegoldia magna* was found to be involved in high-grade squamous intraepithelial neoplasia and cervical cancer (Murphy and Frick, 2013; So et al., 2020; Zhou et al., 2020). *Sporolactobacillus* is similar to *Lactobacillus* in metabolic function and has even been commercialized as a probiotic. Depleted *Sporolactobacillus* may indicate an imbalanced vaginal environment that promotes the growth of pathogenic bacteria (Wei et al., 2020). In brief, significant decreases in probiotics and increases in pathogenic bacterial species were shown among UF subjects, indicating their reduced ability to maintain homeostasis and the increased risk of disease.

Network analysis is a promising and increasingly used approach that involves analyzing and understanding complex systems and emerging phenomena, including understanding functions of the ecosystems, such as stability and resilience (Greenbaum et al., 2019). Analysis of the co-occurrence network of microbiome revealed that microbial networks were composed of tightly connected nodes and formed a kind of “small world” topology. As shown in previous studies, ecological dynamics of the vaginal microbiome play a crucial role in health and disease (Ma and Ellison, 2021). Our network analysis demonstrated differences in the microbial interaction network between the patients and healthy people at different locations. In these two sites of the genital tract, the microbial network in patients with UFs presented lower connectivity and complexity, suggesting that the microbiome found in UFs could be less stable (Naqvi et al., 2010). Therefore, dysbiosis of the microbiome in the cervix and vagina is reflected not only in the changes of microbial relative abundance at the different taxonomic level, but also in the alteration of relationships within microbial interactions.

However, certain limitations of this study should also be considered. Several potential risk factors, such as unhealthy habits and genetic susceptibility, were not described in detail. More completed microbial community information and precise functional genes cannot be provided by 16S rRNA sequencing, although the method has the advantage of easy analysis and low cost. Thus, metagenomic, metatranscriptomics, and/or metabolomic technologies are supposed to conduct further study of microbial communities comprehensively and identify species and strains with higher resolution and confidence. Likewise, no precise mechanism is involved in this study, such as host hormone levels, metabolism, and immune regulation. Therefore, further studies are required to clarify whether the association is causal, whether dysbiosis of the female reproductive tract leads to UFs or whether the disease renders dysbiosis in the vagina and cervix. Moreover, microbial environment of the whole female reproductive tract may not be provided or reflected by vagina and cervical canal, which is closely related to the systemic status. However, it is health-threatening and unethical for healthy women and UF patients who choose minimally invasive treatment to take samples from uterine cavities. Given the ethnic variance, it is reasonable to further broaden the scope of the study population in different ethnic and racial groups to comprehensively identify the microbial alterations in the UFs, thus providing the possibility of geographically tailored microbiome-based therapeutic strategies.

In summary, our preliminary study can provide distinct evidence of the imbalance of vaginal and cervical canal microbiomes in UF patients and serve as a good starting point to narrow down the candidate pathogens for subsequent assays. Our results can lay the foundation for subsequent studies on the role of reproductive tract microbiome in the UF etiology. More studies are needed to analyze the mechanism of how pathogenic bacteria and microbial dysbiosis might affect UFs.

## Data availability statement

The raw data reported in this paper have been deposited in the Gene Expression Omnibus 291 (https://www.ncbi.nlm.nih.gov/geo/), under accession number GSE197904.

## Ethics statement

The studies involving human participants were reviewed and approved by The Third Xiangya Hospital of Central South University and performed under the relevant guidelines and regulations (IRB number 22224). The studies were conducted in accordance with the local legislation and institutional requirements. The participants provided their written informed consent to participate in this study.

## Author contributions

DX and ZY conceived the study. XM and HC performed the experiments and analyzed the data. XM, HC, XP, and XZ wrote and edited the final manuscript. All authors contributed to the article and approved the submitted version.
