# Supplementary material for: Dysbiosis of vaginal and cervical microbiome is associated with uterine fibroids
Source: Front Cell Infect Microbiol. 2023 Sep 6;13:1196823. doi: 10.3389/fcimb.2023.1196823 (PMC10513091; doi:10.3389/fcimb.2023.1196823)
Supplement: Supplementary file 1 [file DataSheet_1.docx]

**Supplementary Material**


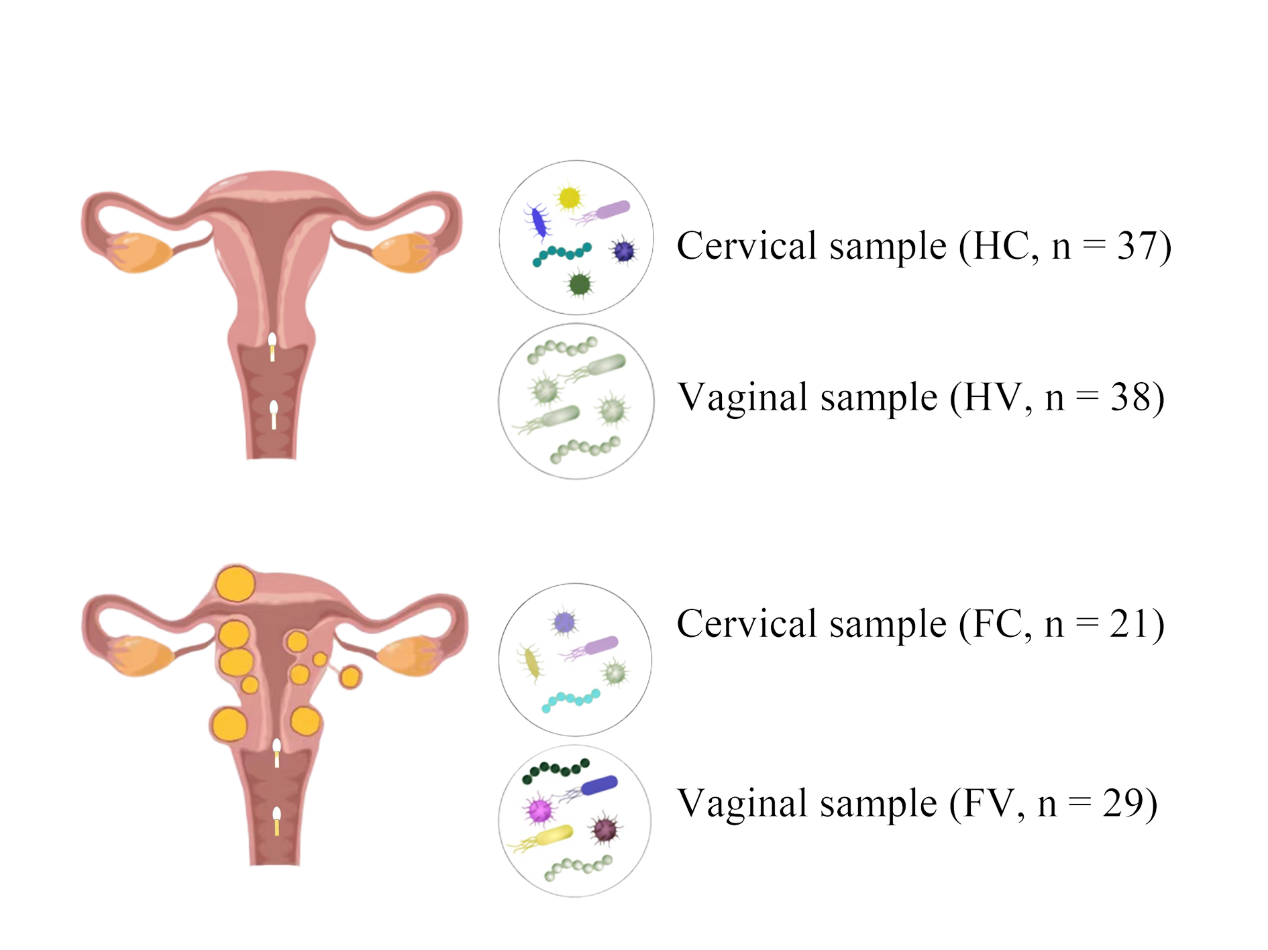


**Fig. S1** **Sample collection.** Diagram of the female reproductive tract showing uterine fibroids and sampling sites within the cervical canal and middle vagina sites.

**
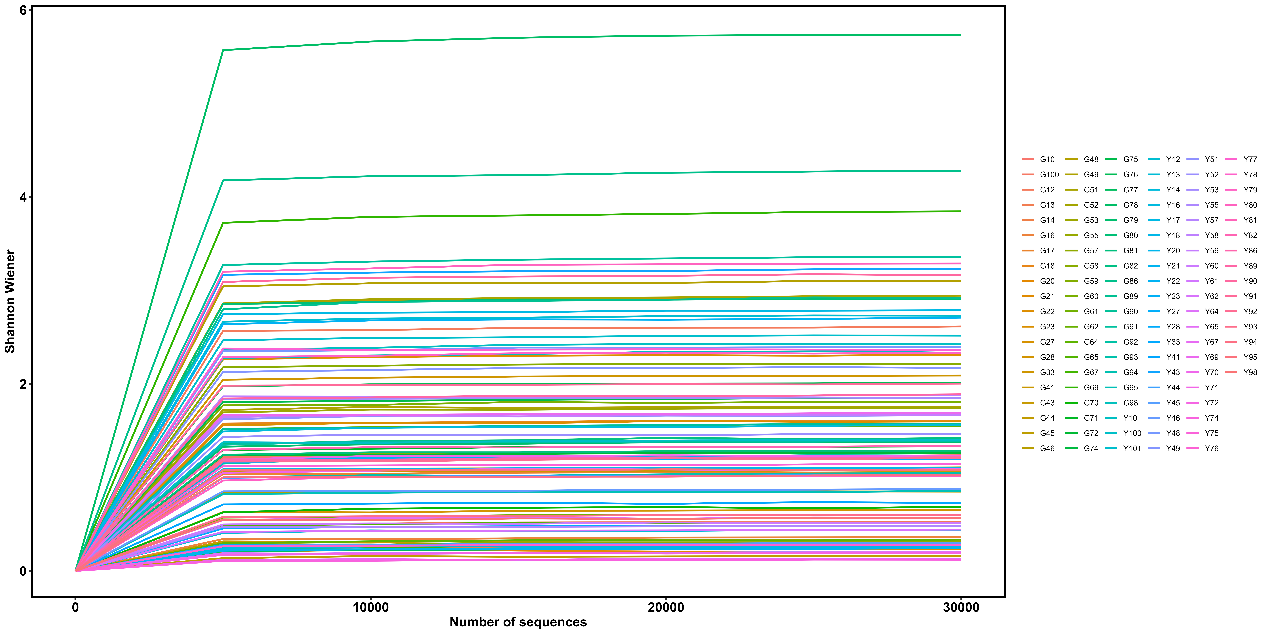
Fig. S2 The species rarefaction curves of all samples.**

**Table S1. Clinical data of 29 patients with uterine fibroids.**

| **Clinical characteristics** | **UFs Patients (n = 29)** |
| --- | --- |
| **Number of fibroids, n (%)** |  |
| Single | 16 (55.17%) |
| Multiple | 13 (44.83%) |
| **Number of recurrences, n (%)** | 3 (10.34%) |
| **Location, n (%)** |  |
| Submucosal | 4 (13.79%) |
| Intermuscular | 17 (58.62%) |
| Subserous | 5 (17.25%) |
| Submucosal & Intermuscular | 3 (10.34%) |
| **Clinical symptoms, n (%)** | 13 (44.83%) |
| **Special pathological type, n (%)** | 1 (3.45%) |
